# *Cissampelos pareira* Linn: Natural Source of Potent Antiviral Activity against All Four Dengue Virus Serotypes

**DOI:** 10.1371/journal.pntd.0004255

**Published:** 2015-12-28

**Authors:** Ruchi Sood, Rajendra Raut, Poornima Tyagi, Pawan Kumar Pareek, Tarani Kanta Barman, Smita Singhal, Raj Kumar Shirumalla, Vijay Kanoje, Ramesh Subbarayan, Ravisankar Rajerethinam, Navin Sharma, Anil Kanaujia, Gyanesh Shukla, Y. K. Gupta, Chandra K. Katiyar, Pradip K. Bhatnagar, Dilip J. Upadhyay, Sathyamangalam Swaminathan, Navin Khanna

**Affiliations:** 1 Department of Microbiology, New Drug Discovery Research, Ranbaxy Research Laboratories, Gurgaon, Haryana, India; 2 Recombinant Gene Products Group, International Centre for Genetic Engineering and Biotechnology, New Delhi, India; 3 Department of Pharmacology, New Drug Discovery Research, Ranbaxy Research Laboratories, Gurgaon, Haryana, India; 4 Department of Pharmacology, All India Institute of Medical Sciences, Ansari Nagar, New Delhi, India; University of Texas Medical Branch, UNITED STATES

## Abstract

**Background:**

Dengue, a mosquito-borne viral disease, poses a significant global public health risk. In tropical countries such as India where periodic dengue outbreaks can be correlated to the high prevalence of the mosquito vector, circulation of all four dengue viruses (DENVs) and the high population density, a drug for dengue is being increasingly recognized as an unmet public health need.

**Methodology/Principal findings:**

Using the knowledge of traditional Indian medicine, Ayurveda, we developed a systematic bioassay-guided screening approach to explore the indigenous herbal bio-resource to identify plants with pan-DENV inhibitory activity. Our results show that the alcoholic extract of *Cissampelos pariera* Linn (*Cipa* extract) was a potent inhibitor of all four DENVs in cell-based assays, assessed in terms of viral NS1 antigen secretion using ELISA, as well as viral replication, based on plaque assays. Virus yield reduction assays showed that *Cipa* extract could decrease viral titers by an order of magnitude. The extract conferred statistically significant protection against DENV infection using the AG129 mouse model. A preliminary evaluation of the clinical relevance of *Cipa* extract showed that it had no adverse effects on platelet counts and RBC viability. In addition to inherent antipyretic activity in Wistar rats, it possessed the ability to down-regulate the production of TNF-α, a cytokine implicated in severe dengue disease. Importantly, it showed no evidence of toxicity in Wistar rats, when administered at doses as high as 2g/Kg body weight for up to 1 week.

**Conclusions/Significance:**

Our findings above, taken in the context of the human safety of *Cipa*, based on its use in Indian traditional medicine, warrant further work to explore *Cipa* as a source for the development of an inexpensive herbal formulation for dengue therapy. This may be of practical relevance to a dengue-endemic resource-poor country such as India.

## Author Summary

India represents ~50% of the global population estimated to be at risk of dengue. Severe dengue, which is potentially fatal, correlates with very high virus load, reduction in platelet counts and haemorrhage. Antiviral therapy to reduce high virus load may be beneficial in attenuating disease severity. We have explored Indian traditional medicine, Ayurveda, to identify plants that could be a source of dengue inhibitory activity. We show that an alcoholic extract prepared from *Cissampelos pareira* Linn inhibited the replication of dengue viruses in living cells in culture and protected mice against dengue infection. It also showed antipyretic and anti-inflammatory effects. Importantly, this extract did not show any toxic effects in rats and did not affect platelets and red blood cells. This observation is critical as dengue fever is commonly treated with antipyretics. In a dengue-endemic resource-poor country as India, the *C*. *pareira* plant may serve as a source for the development of an inexpensive herbal formulation against dengue.

## Introduction

Dengue disease is a major public health concern around the world. It is spread to humans through the bite of *Aedes* mosquitoes which serve as carriers of the disease-causing viruses. There are four serotypes of dengue viruses (DENV-1, -2, -3 and -4), belonging to the family *Flaviviridae* [1], which are prevalent in more than a hundred mosquito-infested countries in the tropical and sub-tropical regions of the globe [2, 3]. According to recent estimates, there are annually ~400 million infections around the world and a fourth of these are associated with symptomatic dengue illness [3]. Symptomatic dengue illness can range from mild dengue fever (DF) to severe and potentially fatal dengue haemorrhagic fever (DHF) and dengue shock syndrome (DSS) [4]. In DF, the appearance of virus in the serum coincides with fever onset, with titers reaching 10^6^ infectious units/ml, at the peak of the febrile phase. In DHF/DSS patients, the viremia can escalate by 1–2 logs [5, 6]. This is accompanied by potentially fatal manifestations of thrombocytopenia, haemorrhage, vascular leakage and shock [1, 2, 7]. Unless hospitalized and given supportive care and fluid replacement, DHF/DSS can be associated with high case fatality rates [4]. Controlling the spread of dengue continues to be an intractable problem due to the inability to eradicate the vector mosquitoes and the lack of safe, potent preventive vaccine [8, 9]. Despite numerous hurdles, persistent efforts over the years have resulted in the development of several live attenuated dengue vaccine candidates [9], one of which has undergone phase III clinical testing [10–12] and is expected to be licensed in the near future. The challenges being faced in dengue vaccine development [13] have emphasized the need for antiviral drugs and spurred new efforts in this direction [14–16].

In regard to drugs, a majority used in treating human illness [17] and several in clinical development [18] can be traced to natural origins. India has a rich herbal repertoire which is used in traditional ethnomedicine. The current work was undertaken with the objective of exploring the possibility of identifying anti-DENV activity that may be associated with indigenous plants in India. To choose plants likely to provide useful leads, the study utilized knowledge from traditional Indian medicine, Ayurveda, a specific branch of Indian ethnomedicine [19]. Ayurveda prescribes medicines based on various herbs for a variety of illnesses. Though dengue as such is not described in Ayurvedic literature, there are illnesses identified by symptoms that can be correlated with some of the clinical manifestations of dengue disease. Plants shortlisted on this basis from the vast indigenous herbal bio-resource available in India were screened for anti-DENV activity. This work describes the screening assays that were set-up to identify anti-DENV activity and presents data identifying two plants that manifested inhibitory potency against all 4 DENVs. One of these, chosen for further study to assess its *in vivo* efficacy and clinical relevance, was found to be a promising candidate for further development as a pan-DENV inhibitory formulation.

## Methods

### Cells and viruses

The mosquito cell line C6/36, the monkey kidney cell lines LLCMK_2_ and Vero, and human hepatoma cell line HepG2 were from American Type Culture Collection, Virginia, U.S.A. C6/36 cells were maintained in Leibovitz L-15 medium supplemented with 0.03% tryptose phosphate broth and 10% heat-inactivated fetal calf serum (ΔFCS) in a CO_2_-free incubator at 28°C. The monkey kidney cells were maintained in Dulbecco’s Modified Eagle medium (DMEM), supplemented with 10% ΔFCS, in a 5% CO_2_ humidified incubator, at 37°C. For HepG2 cells, DMEM was replaced by Roswell Park Memorial Institute (RPMI) medium. The remaining conditions were the same. Representatives of the four DENV serotypes (strain and accession numbers are indicated in parentheses) used in this study were: DENV-1 (Nauru Island, U88535); DENV-2 (New Guinea C, AF038403), DENV-3 (H87, M93130) and DENV-4 (Dominica, M14931). These were propagated in C6/36 cells and titrated on LLCMK_2_ cells using a standard plaque assay as described below.

### Plants and preparation of extracts

Plants shortlisted for screening are indicated in Table 1. These were procured through local suppliers and identified and authenticated by the resident herbal taxonomist at Ranbaxy Research Laboratories, Gurgaon, India. Extracts were prepared using part(s) of the plants used in conventional Ayurvedic preparations. In each case, three different extracts, methanolic, hydroalcoholic and aqueous, were prepared using methanol, methanol: water (1:1), and water, respectively. Plant parts were washed, shade-dried and pulverized prior to extraction. To prepare extracts, ~100 g plant material was extracted three times with 500 ml extraction solvent at boiling point for 3 hours, each time. The three extractions were pooled, filtered and concentrated at low pressure and temperature, and dried in a vacuum oven at room temperature (RT) for 16–18 hours. The resultant material was dissolved in dimethylsulfoxide (DMSO), at 20mg/ml and stored at 4°C.

**Table 1:** Screening of plant extracts for anti-DENV activity.

No.	Name of Plant	Part used^*a*^	Antiviral Activity^*b*^
1	*Aconitum heterophyllum*	Rhizome	-
2	*Andrographis paniculata*	Whole plant	-
3	*Azadirachta indica*	Bark	-
4	*Cedrus deodara*	Resin	-
5	*Cissampelos pareira*	Aerial part	+
6	*Coptis teeta*	Root	+
7	*Cyperus rotundus*	Rhizome	-
8	*Desmodium gangeticum*	Whole plant	+
9	*Enicostemma littorale*	Whole plant	-
10	*Fumaria indica*	Whole plant	+
11	*Ocimum sanctum*	Whole plant	-
12	*Phyllanthus amarus*	Whole plant	+
13	*Picrorhiza kurroa*	Rhizome	±
14	*Premna mucronata*	Bark	+
15	*Swertia chirayata*	Whole plant	-
16	*Terminalia chebula*	Fruit	+
17	*Tinospora cordifolia*	Stem	+
18	*Aloe vera*	Leaf	-
19	*Citrullus colocynthis*	Root	-

^*a*^Methanolic extracts were prepared using parts of the plant mentioned in Ayurvedic literature

^*b*^Antiviral activity against DENV-3 screened using Type-1 assay. “+” indicates active extract (IC_50_ <25μg/ml); “±” indicates borderline extracts (IC_50_ = ~25μg/ml); “-” indicates inactive extract (IC_50_ >25μg/ml)

### Plaque assay

The titer of infectious DENV in virus stocks and in culture supernatants (in the type-3 screening assay, see below) were determined using a standard plaque assay as described previously [20]. Briefly, LLCMK_2_ monolayers in 6 well plates were infected in duplicate with serial 10-fold dilutions (prepared in DMEM+2%ΔFCS) of the virus-containing samples (250μl/well). Mock-infections were performed in parallel using an equivalent volume of virus diluent alone. Two hours later, the infected monolayers (after aspirating off the virus inoculum) were overlaid with DMEM+6%ΔFCS containing 1% methyl cellulose (2 ml/well), and incubated for 6 days (37°C, 5% CO_2_). On day 6 post-infection, the overlay was removed and the cells fixed with 4% formaldehyde solution (1 ml/well). Fixed cells were washed and stained with 0.05% (w/v) crystal violet solution in 20% ethanol. Revealed plaques were counted to determine the virus titre, expressed as plaque-forming units (PFUs)/ml.

### Cell-based bioassays for antiviral screening

The initial antiviral screening assay, designated as the type-1 assay, was based on the plaque reduction neutralization test (PRNT) described earlier for determining DENV-neutralizing antibody titres in sera [20]. LLCMK_2_ cells were seeded in 24-well plates (5x10^5^ cells/well), a day in advance. DENV-1, -2, -3 and -4 (100 PFU each) were separately pre-incubated with serial dilutions of herbal extracts (corresponding to 0–100 μg/ml final concentration) in 300 μl volume, at 4°C overnight. The pre-incubation mixture was diluted with an equal volume of medium (DMEM+ 2% ΔFCS) and used to infect LLCMK_2_ cells (3 wells for each concentration at 200 μl/well) in the 24-well plate. After 2 hours of adsorption in the incubator (37°C, 5% CO_2_), infected cells were overlaid with methylcellulose-containing growth medium and processed thereafter as described for the plaque assay above. To assess any potential cytotoxicity, cells were exposed to the herbal extracts (in the same concentration range) in the absence of DENV infection. Additional control experiments, run in parallel, included cells which were either mock-infected (negative control) or infected with DENV in the absence of herbal extract (positive control). The half-maximal inhibitory concentration (IC_50_ value) for each herbal extract against each DENV serotype, with reference to the positive control which represented 100% infection (or 0% inhibition), was defined as the concentration of herbal extract, in μg/ml, resulting in 50% inhibition of the plaque count.

In the type-2 screening assay, LLCMK_2_ cells in 24-well plates were infected with DENVs (moi = 0.002) without pre-incubating with the herbal extracts. After 2 hours of adsorption, the virus inoculum was aspirated, the monolayer rinsed, and then fed with complete medium containing the herbal extracts (corresponding to 0–200 μg/ml final concentration). After 24 hours of exposure to the extract, the monolayer was aspirated and overlaid with growth medium containing methyl cellulose and plaques developed 6 days later as above.

The type-3 assay was done using Vero cells, as these cells secrete the viral antigen NS1 and the infectious virions efficiently into the culture medium upon DENV infection. The assay design was similar to the type-2 assay, except that following sequential exposure of cells to DENV and herbal extract, cells were fed with liquid growth medium, instead of the methylcellulose overlay. Aliquots of the culture supernatant were withdrawn at periodic intervals up to 9 days for estimation of NS1 antigen levels (using a commercial ELISA kit) and virus titres (by plaque assay, as described above).

### Determination of cytotoxicity of *Cipa* extract

Cytotoxicity was evaluated in two cell lines, LLCMK_2_ (in which the antiviral activity of the extracts were assayed) and HepG2, a commonly used liver cell surrogate for *in vitro* cytotoxicity testing. Cells seeded in 96-well plates were exposed to a wide concentration range of *Cipa* extract (1–200 μg/ml) for 3 days. Cell viability was assessed using a commercial MTT (3-[4,5-dimethylthiazol-2-yl]-2,5-diphenyltetrazolium bromide) assay kit (Sigma, cat. # M5655) with reference to control cells that were not exposed to the extract. The half-maximal cytotoxic concentration (CC_50_ value) for the herbal extract, with reference to the positive control (untreated cells) which represented 100% cell viability (or 0% cytotoxicity), was defined as the concentration of herbal extract, in μg/ml, resulting in 50% cytotoxicity. Selectivity index (SI) of an extract is defined as the ratio of CC_50_ to IC_50_ values obtained using the LLCMK_2_ cell line.

### Effect of pre-incubation time and virus dose on the anti-DENV activity of *Cipa* extract

To assess if the duration of pre-incubation of the *Cipa* extract with DENV influenced antiviral activity in the type-1 assay format, pre-incubation times ranging from 0–24 hours were tested using ~50 PFUs of DENV-3.

To assess the effect of the size of the DENV dose on the anti-DENV efficacy of *Cipa* extract in the pre-incubation step (at 4°C, overnight), type-1 assays were performed using DENV-3 ranging from 50 to 5000 PFUs. Each dose of DENV-3 was assayed against *Cipa* extract ranging in concentration from 0–200 μg/ml.

### Evaluation of *in vivo* efficacy of *Cipa* extract in the AG129 mouse model

To prepare a stock of challenge virus capable of lethal infection in AG129, we used a previously published strategy [21, 22] with some modifications. Essentially DENV-2 (NGC) was alternately passaged between AG129 (intracranial inoculation of 10^6^ PFU) and C6/36 cells in tissue culture. After 4–5 such cycles of passaging, the virus was tested in AG129 mice to determine the minimum lethal dose (MLD) by i.p. injections. MLD is defined as the dose that can cause clinical symptoms and 90–100% death 3–4 weeks post challenge. The challenge virus stock thus obtained was titrated, aliquotted and stored in liquid N_2_ until use. To test protective efficacy of *Cipa* extract, AG129 mice (9–12 weeks old, 20–24 g body weight) were challenged with 10^6^ PFU (per mouse, 0.4 ml, i.p) of the challenge DENV-2 stock described above. Challenged mice were divided into groups (*n* = 6) and treated orally with vehicle alone (0.25% methyl cellulose) or with two different doses of *Cipa* extract (at 125 mg and 250 mg/kg body weight). The methanol in the *Cipa* extracts administered to mice was removed completely by evaporation. The resultant methanol-free *Cipa* paste was thoroughly re-suspended in 0.25% methyl cellulose water and administered orally to infected mice. The volume of the oral dose was adjusted in accordance with the body weight of each animal (10 ml/Kg/dose) and administered by a trained veterinarian using a specially designed mouse feeder needle fitted with a graduated 1 ml disposable syringe. The treatment was initiated 2 hours post-infection and continued twice daily for 5 consecutive days. Animals were monitored twice daily for a period of 35 days for clinical symptoms and mortality. A control (sham) group that was not virus-challenged, but which received *Cipa* extract (250 mg/kg), was also tested in parallel. At the end of the experiment, the survival data was used to plot Kaplan Meier survival curves and analysed by the log rank test (Mantel-Cox) test for statistical significance using GraphPad Prism 5 software.

### Determination of effect of paracetamol on *Cipa* extract *in vitro* and *in vivo*

Interaction between paracetamol and *Cipa* extract was assessed *in vitro* using type-1 assay format as follows. DENV-3 (~50 PFUs) and *Cipa* extract (ranging in concentration from 0–50 μg/ml) were pre-incubated overnight at 4°C in a volume of 100 μl, and used to infect LLCMK_2_ cells in 24-well plates. Parallel infections were set up using pre-incubation mixtures containing paracetamol (1–100 μg/ml), in addition to DENV and *Cipa* extract. As before, mock-infections and DENV only infections (in the absence of *Cipa* and paracetamol) were also set up and analysed in parallel.

The *in vivo* effect of *Cipa* extract in the presence and absence of paracetamol was assessed using the Wistar rat pyrexia model. Wistar rats (weighing 180–220g) of either sex were used. Basal temperature of the rats was measured using a digital rectal thermometer (Experimetria Ltd., Hungary) and then injected subcutaneously (in the intra-scapular region) with 20% brewer’s yeast (10 ml/kg body weight) and allowed to fast overnight with free access to water. At 18 hours post-injection, rectal temperatures were recorded again to identify animals that registered ≥0.7°C rise in body temperature for inclusion in the study. Groups (*n* = 7–9) of febrile rats were orally administered paracetamol (200 mg/kg), or *Cipa* extract (200 mg/kg) or both. Rats in the control group received just the vehicle (0.5% methyl cellulose). This was followed by recording of rectal temperature for 3 hours at 30 minute intervals.

### Effect of *Cipa* extract on erythrocytes and platelets *ex vivo* and *in vivo*

For *ex vivo* studies, human blood was collected from healthy adult donors after informed consent. Erythrocytes were pelleted down in a centrifuge (1500*x*g, 5 minutes) from freshly collected heparinized blood, rinsed thoroughly with PBS (pH 7.4), and used to make a 1% cell suspension in PBS. *Cipa* extract ranging in concentration from 12.5 to 400 mg/L was added to the erythrocyte suspension and incubated at 37°C for 1 hour. After this, the samples were spun down, and the absorbance of the supernatant measured at 576 nm to determine the extent of erythrocyte lysis. Controls wherein erythrocytes were incubated with buffer alone (0% lysis), DMSO alone (*Cipa* solvent) and 0.1% Triton X-100 (100% lysis) were processed in parallel. Basal platelet count in freshly collected heparinized blood and in blood pre-incubated with DMSO (vehicle) or *Cipa* extract (2–10 μg/ml) for different durations (1–4 hours) was determined using a Beckman Coulter hematology analyser.

For *in vivo* studies, four groups (n = 5) of Wistar rats were fasted overnight and administered orally with vehicle (0.25% methyl cellulose) or *Cipa* extract, at three different dosages (100, 300 and 1000 mg/kg body weight). Blood was collected just before *Cipa* extract administration (0 hour) and at 1 and 4 hours post-administration. Hematology parameters were measured using ADVIA-120 hematology analyser.

### Cytokine release assay

Human peripheral blood mononuclear cells (PBMCs) were obtained as follows. Freshly collected heparinized blood was diluted with an equal amount of RPMI 1640 medium and layered on Ficoll Hypaque 1077 and centrifuged at 2,500 rpm for 25 minutes at RT. The upper layer was discarded and the fluffy layer at the interphase was harvested, rinsed and re-suspended in RPMI 1640 at 5x10^5^ cells/ml. Freshly collected PBMCs were seeded in 96-well plates (10^5^ cells/well) and treated with *Cipa* extract at different dilutions (in RPMI 1640), followed by 30 minutes incubation at RT on a rotary shaker (200 rpm). Next, wells were treated with 50μl (4μg/ml) lipopolysaccharide (Sigma Cat. # L2654) and allowed to incubate for a further 30 minutes at RT. The volume per well was made up to 200 μl using RPMI+10%FCS and the plates incubated overnight at 37°C in a CO_2_ incubator. Negative controls (no lipopolysaccharide treatment) were run in parallel. The plates were centrifuged (3000 rpm, 10 minutes) to obtain clarified supernatants for TNF- α (e-Bioscience, cat # BMS223/4TEN,) and IL-1β (BD Bioscience, cat # 557953) determinations using commercial ELISA kits as per the vendors’ instructions.

### Toxicology

Groups of 5 adult Wistar rats were orally administered 4 ml 0.25% methyl cellulose (vehicle)/kg or 4 ml vehicle containing 400 mg to 2000mg *Cipa* extract/kg, once daily for 7 days (in accordance with OECD guidelines- 407). During this period, food intake, body weight, and clinical signs were monitored daily. At the end of the experiment, animals were euthanized, followed by determination of hematological (Hb, WBC count, RBC count, platelet count and hematocrit) and biochemical (SGOT, SGPT, total protein, serum albumin, total cholesterol, urea, creatinine and random sugar) parameters. Necropsy was performed. Organ weights were recorded and histopathology was done.

### Ethical clearance

Human blood from healthy volunteers was collected after written informed consent in strict accordance with approved guidelines of the Human Ethics Committee, Ranbaxy Laboratories, which approved the ‘Clinical protocol for collection of blood samples from healthy adult human subjects for screening of potential new chemical entities to inhibit cytokine release and other *in vitro* parameters in an assay system’ through Ethical Approval number: 5001_cok_11. All animal experiments were performed in strict accordance with guidelines specified by the Committee for the Purpose of Control and Supervision of Experiments on Animals (CPCSEA) of the Government of India. Animal experimental protocols were approved by the Institutional Animal Ethics Committee (IAEC) of Ranbaxy Research Laboratories, India through Approval number 151/07.

## Results

### Design of screening assays

**Fig 1:**
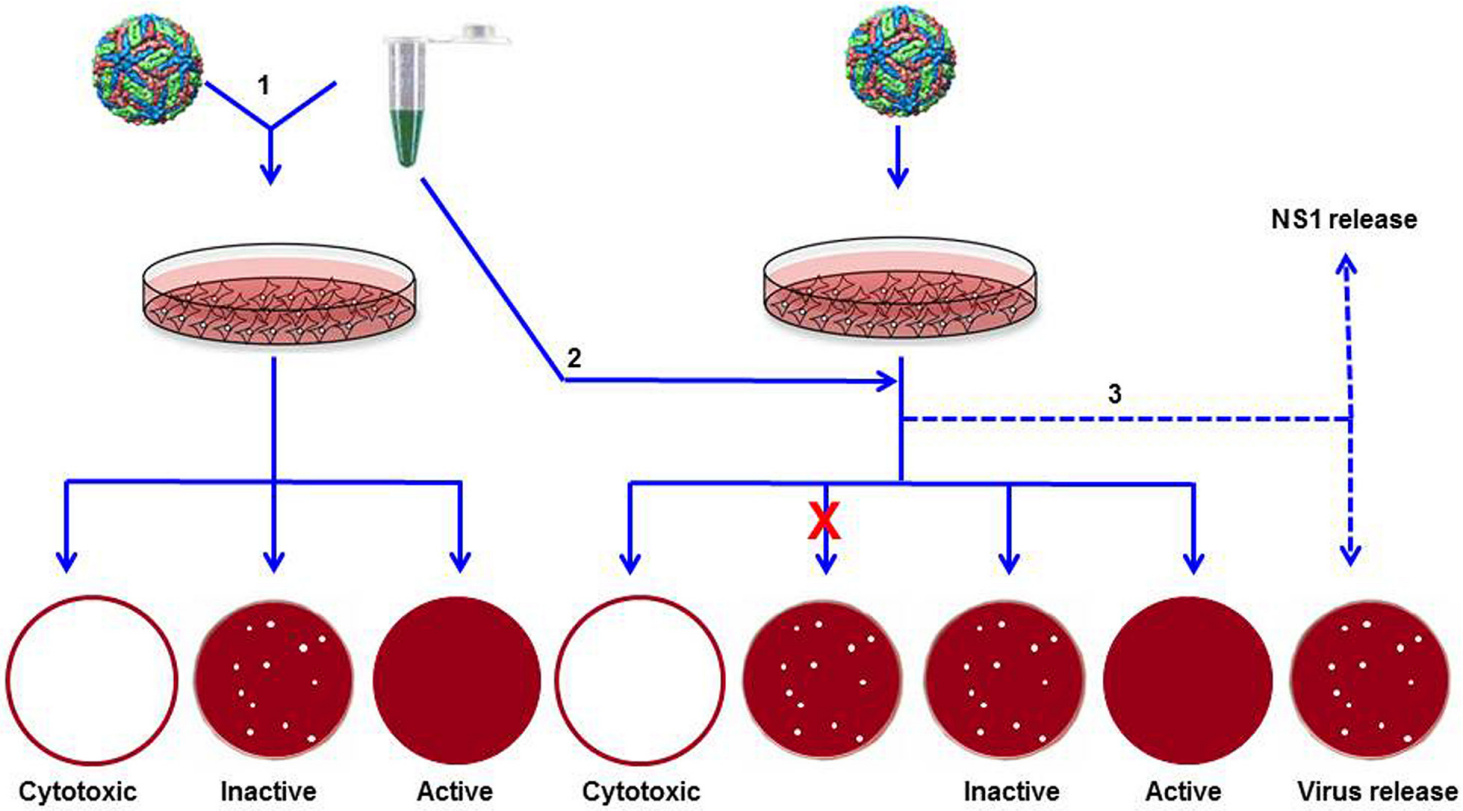
Schematic representation of the antiviral screening assays. An outline of the three types of screening assays (indicated by Arabic numbers 1, 2 and 3) is shown. The multi-coloured sphere represents DENV and the Eppendorf tube with green liquid represents the herbal extract. These two were pre-incubated (1) before addition to the monolayer or added sequentially (2, 3) to the monolayer. In assays 1 and 2, the treated-monolayers were overlaid with methyl cellulose-containing growth medium. Shown at the bottom are the possible outcomes of the type 1 and 2 assays. The ‘x’ mark denotes failure of entry into cells. In assay 3, liquid growth medium was added instead of the methyl cellulose overlay, and followed by analysis of NS1 and virus released into the culture supernatant.

Three bioassays were developed to identify herbal extracts with potential DENV-inhibitory activity, as shown schematically in Fig 1. The type-1 assay was designed to identify herbal extracts that had the ability to block DENV from entering susceptible cells. As a standardized reference is not available to assess the efficacy of DENV inhibitory potency, PRNT data of monoclonal antibodies (mAbs) were used to decide on the cut-off value in the type-1 assay. For example, two pan-DENV mAbs, 4G2 and DB13-19, show half maximal inhibition of DENV-2 plaque counts at 11 and 33 μg/ml, respectively [23]. Based on this observation, an extract which showed an IC_50_ value ≤ 25μg/ml, a value which falls between these two, was designated to be active as a DENV inhibitor. Considering that this value is for a crude multicomponent extract, it represents a stringent cut-off. In the context of antiviral screening, this assay had the following possible outcomes: the extract could be cytotoxic (compromised monolayer), inactive (no reduction in plaques in comparison to control infection), or active (reduced number of plaques). However, the type-1assay is not likely to necessarily reveal if the active extracts also possessed the ability to inhibit post-entry steps in the DENV life cycle.

The type-2 assay was designed to assess the capacity of the herbal extracts to inhibit DENVs within the cell. Therefore, DENV infection preceded exposure to the herbal extract. As the herbal extract must be internalized before it exerts any inhibitory effect, we decided to use higher amounts of the extract. Accordingly, the cut-off in the type-2 assay was increased to 150μg/ml. Possible outcomes in the type-2 assay are as follows: once again, the extract could be cytotoxic. If not, it may be ineffective due to one of two reasons: the extract failed to enter the cells, or it was ineffective despite successful entry. The final possibility is that the extract would reduce the plaque count, indicating antiviral activity. The type-3 assay was designed to monitor inhibition based on reduction in viral antigen production and virus yields from DENV-infected cells following treatment with herbal extracts. The amounts of viral NS1 antigen and infectious virus released into the culture supernatant were measured over time to gauge the magnitude of inhibition.

In order to identify an effective herbal extract against dengue, it was reasoned that it must target all four DENVs. Further, it would be desirable to be able to inhibit the viruses both before as well as after entry into susceptible cells. Towards this end, all extracts were screened against a single DENV serotype in the type-1 assay, followed by screening of the active extracts against the remaining three serotypes. Next, extracts manifesting pan-DENV inhibitory potential in the type-1 assay were progressed to screening in the type-2 assay. Extracts identified as active against all 4 DENVs in the type-1 and -2 assays were tested in type-3 assay to assess their ability to reduce virus titres by ≥ ten-fold.

### Identification of plant extracts with pan-DENV inhibitory activity

Nineteen plants described in Ayurvedic literature as being beneficial in the treatment of illnesses with dengue-like symptoms were shortlisted for bioassay guided screening (Table 1). Initial screening showed that only the methanolic extracts manifested antiviral activity when assayed against DENV-2 or DENV-3. The hydroalcoholic and aqueous extracts of all 19 plants selected for the study did not manifest any antiviral activity when tested against these two DENV serotypes (IC_50_ >>100μg/ml). As a result, all subsequent studies were carried out using the methanolic extracts.

Methanolic extracts were prepared from each of these and screened against DENV-3 in a type-1 assay. Extracts that manifested IC_50_ values below the pre-designated cut-off were scored as positive and the remaining as negative for antiviral potency. The results are summarized in Table 1. This screening assay revealed 8 of 19 extracts to possess definite anti-DENV-3 inhibitory activity, while one manifested borderline inhibition. These 9 extracts were then screened against the remaining three DENV serotypes as well in the type-1 assay. These results, shown in Table 2, revealed that 4 of the 9 anti-DENV-3 extracts manifested potent inhibitory activity against the remaining three DENV serotypes as well. The rest were effective at least against two other DENV serotypes. When all these 9 were tested in a type-2 assay against DENV-3 to identify extracts that may inhibit the virus after its entry into cells, two extracts, one obtained from *Cissampelos pareira* Linn, and the other from *Phyllanthus amarus*, were effective in curbing DENV-3 (IC_50_ values <150 μg/ml). Extending the type-2 assay to DENV-1, -2 and -4 revealed that both these extracts possessed the ability to inhibit all four DENVs even after their entry into cells (Table 3). While *Phyllanthus amarus* has been documented to possess antiviral properties against another flavivirus, Hepatitis C virus [24] as well as many other viruses [25], this is the first report of antiviral activity associated with *Cissampelos pareira* Linn. Further studies in this work focused on *Cissampelos pareira* Linn, henceforth designated as *Cipa* for convenience.

**Table 2:** Extracts with Pan-DENV inhibitory activity.

No.	Extract	Antiviral activity (IC_50_, μg/ml)				
		DENV-1^*a*^	DENV-2 ^*a*^	DENV-3 ^*a*^	DENV-4 ^*a*^	DENV-3 ^*b*^
1	*Cissampelos pareira*	11.11	1.97	3.17	1.23	78
2	*Coptis teeta*	11.11	5.04	7.87	33.33	>200
3	*Desmodium gangeticum*	12.5	6.7	14.62	12.5	>200
4	*Fumaria indica*	12.5	7.02	11.32	12.5	>200
5	*Phyllanthus amarus*	3.7	8.33	20.76	11.11	145
6	*Picrorhiza kurroa*	12.5	16.02	27.14	25	>200
7	*Premna mucronata*	12.5	27.9	21.37	12.5	>200
8	*Terminalia chebula*	25	70	11.2	50	>100
9	*Tinospora cordifolia*	50	58.75	16.02	25	>100

^a^Type-1 assay (carried out using all DENV serotypes)

^b^Type-2 assay (carried out using DENV-3 alone)

**Table 3:** Extracts displaying pan-DENV inhibitory activity in Type-2 assay.

No.	Extract	Antiviral activity (IC_50_, μg/ml)			
		DENV-1	DENV-2	DENV-3	DENV-4
1	*Cissampelos pareira*	100	125	78	100
2	*Phyllanthus amarus*	100	133	145	100

### *Cipa* extracts from different geographical locations are similar in composition and antiviral potency

From the perspective of evaluating the availability of starting material, it was considered necessary to ascertain the extent of seasonal and geographical variations in the capacity of the *Cipa* extract to manifest DENV inhibitory activity. To this end, extracts were prepared from *Cipa* plants collected during different seasons from multiple geographical locations within India. Analysis of these extracts revealed that the anti-DENV inhibitory activity is evident in plants collected during the flowering season of April to September [26]. In the experiment, the results of which are summarized in Table 4, *Cipa* extracts were prepared from plants obtained during the flowering season from four different locations, and evaluated using the type-1 assay described above. The data show that *Cipa* extracts manifested pan-DENV inhibitory activity in both assay formats. Further, the observed mean pan-DENV IC_50_ values were not significantly different for all four geographic locations tested. Fractionation of the methanolic extracts with different solvents resulted in nominal increase in antiviral efficacy and a significant loss in yield. Data in Table 4 show that among the solvents tested, methanol extraction is the best with yields ranging from 6–13%. In addition, a preliminary LC-MS analysis of extracts from two different locations (S1 Fig), demonstrates that the two *Cipa* extracts manifest essentially similar profiles. This leads to the conclusion that there is no discernible geographical variation and that the method of extract preparation is standardized and reproducible.

**Table 4:** Profile of anti-DENV activity of extracts from *Cipa* collected from geographically diverse locations.

Extract/Fraction	S. India		N. India		Rajasthan		M. P.	
	IC_50_ ^*a*^ (μg/ml)	Yield^*b*^ (%)	IC_50_ ^*a*^ (μg/ml)	Yield^*b*^ (%)	IC_50_ ^*a*^ (μg/ml)	Yield^*b*^ (%)	IC_50_ ^*a*^ (μg/ml)	Yield^*b*^ (%)
Methanolic	7.12	6	8.01	5.96	7.46	8.62	21.38	13.2
Hexane	2.33	1.37	2.10	1.25	2.89	1.5	2.79	2.15
Chloroform	1.02	0.53	1.05	0.66	1.08	0.25	3.62	0.26
Dichloromethane	2.94	0.61	1.61	0.69	1.72	0.22	6.42	0.3
Ethyl acetate	2.89	2.57	4.07	2.74	2.7	2.93	4.65	3.44
Acetone	1.68	2.11	3.05	2.02	1.88	2.3	2.93	2.78

^*a*^Denotes pan-DENV inhibitory activity (mean of the IC_50_ values targeting each of the four DENV-serotypes)

^*b*^% Yield = (wt. of extract or fraction obtained/wt. of starting material) x100

### *Cipa* extract inhibits secretion of viral antigen and infectious virus

**Fig 2:**
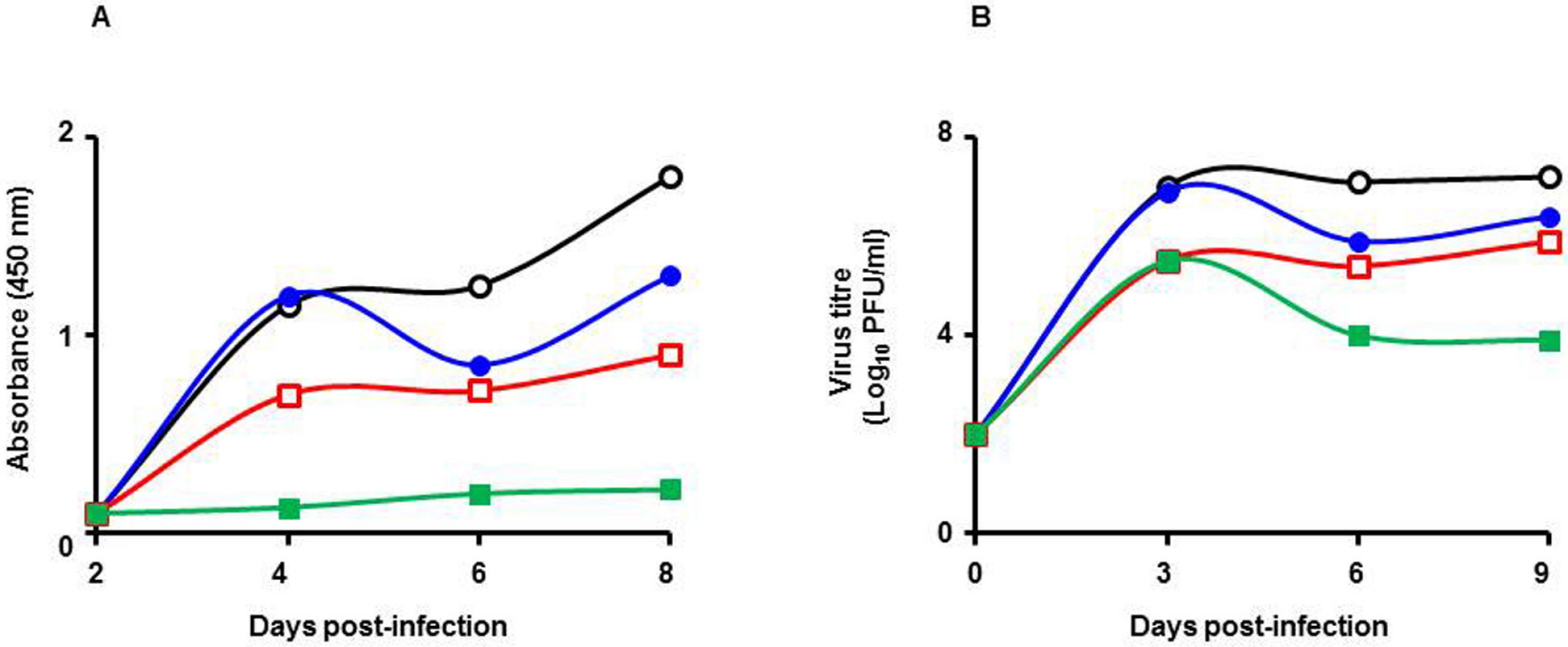
Inhibition of DENV antigen and virus production by *Cipa* extract treatment. Vero cells were used to test the effect of *Cipa* extract on DENV-3 in a type-3 assay format. The figure depicts the kinetics of NS1 antigen (A) and infectious virus (B) released into the culture supernatant in the absence (empty black circles) and presence of *Cipa* extract at 22 μg/ml (filled blue circles), 66 μg/ml (empty red squares) and 200 μg/ml (filled green squares) concentrations.

The kinetics of virus inhibition by *Cipa* extract was analysed in a type-3 assay. Aliquots of the culture supernatant were withdrawn at regular intervals over a period of several days and analysed for the presence of viral NS1 antigen and infectious virus, as shown in Fig 2. In control experiments wherein infected cells were not exposed to *Cipa* extract, NS1 antigen was detectable after day 2 onwards and rising thereafter during the course of the experiment. In parallel experiments, the exposure of cells to *Cipa* extract had a dose-dependent inhibitory effect on NS1 antigen secretion. While the inhibition resulting from exposure to a low dose of *Cipa* extract was manifested after day 4 post-infection, inhibition at higher doses was evident earlier and at relatively higher magnitudes (Fig 2A). In fact, at the highest dose of *Cipa* extract tested in this experiment (100μg/ml), the inhibition of NS1 antigen was near total for the entire duration of the experiment. The inhibition of viral antigen synthesis evident from this experiment suggests that virus production would also be similarly affected. This notion was substantiated by determination of viral titers in the culture supernatants during the course of the above experiment, as shown in Fig 2B. In the control experiment, viral titers increased steadily reaching a plateau at day 3 post-infection. *Cipa* extract lowered viral titers in a dose-dependent manner as seen for NS1 secretion. Thus, at the lowest concentration of *Cipa* extract used, reduction in viral titers became apparent from day 4 onward. Significantly, a small increase in the *Cipa* dosage resulted in >1 log reduction in viral titer as early as day 3 post-infection. At the highest dose of *Cipa* extract tested (100μg/ml), the drop in viral titers was ~2 logs. Importantly, the reduction in viral titers was sustained over a period of several days. Interestingly, the magnitude of inhibition appeared to be greater based on NS1 levels compared to viral titers. The data suggest that the *Cipa* extract may have effects on NS1 antigen synthesis and release that are distinct from its effects on virus replication.

**Fig 3:**
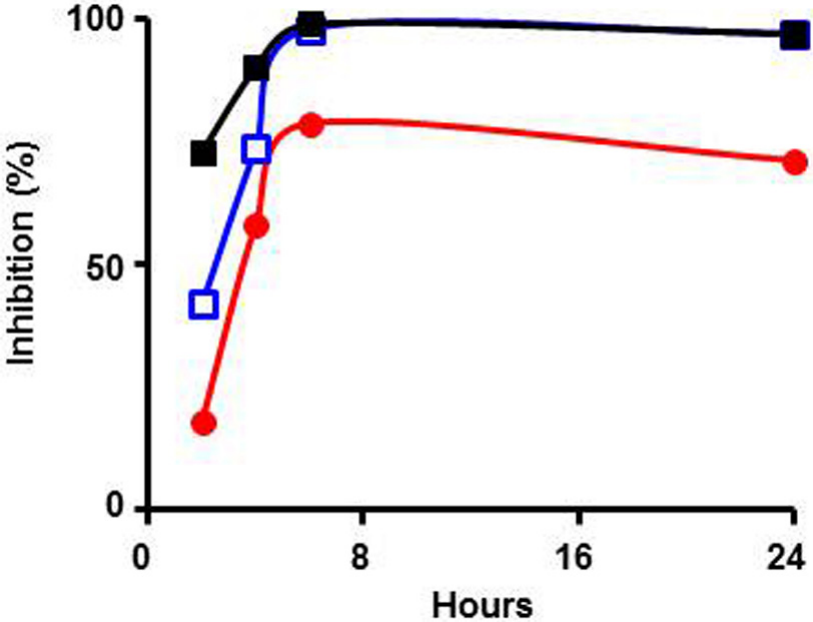
Effect of pre-incubation time on antiviral activity of *Cipa* extract. DENV-3 (50 PFU) was pre-incubated with *Cipa* extract at 11 μg/ml (filled red circles), 33 μg/ml (empty blue squares) and 100 μg/ml (filled black squares) for different durations (2–24 hours) followed by assay of antiviral activity in a type-1 assay.

Next, a complementary experiment was performed wherein DENV-3 was pre-incubated with increasing concentrations of *Cipa* extract for different periods of time before infection (type-1 assay) and overlay. Plaque counts obtained at the end of the experiment revealed a dose- and time-dependent virucidal effect of *Cipa* extract on DENV-3 as depicted in Fig 3. A converse experiment, again in type-1 format, was carried out to determine the inhibitory efficacy of the extract against DENV-3 stocks whose titers varied over 2 logs. The IC_50_ values corresponding to DENV-3 dosage of 50, 500 and 5000 PFUs were, respectively, 9.92, 12.5 and 44.45 μg/ml. This leads to the conclusion that the antiviral potency of *Cipa* extract extends over a wide range of viral loads.

**Fig 4:**
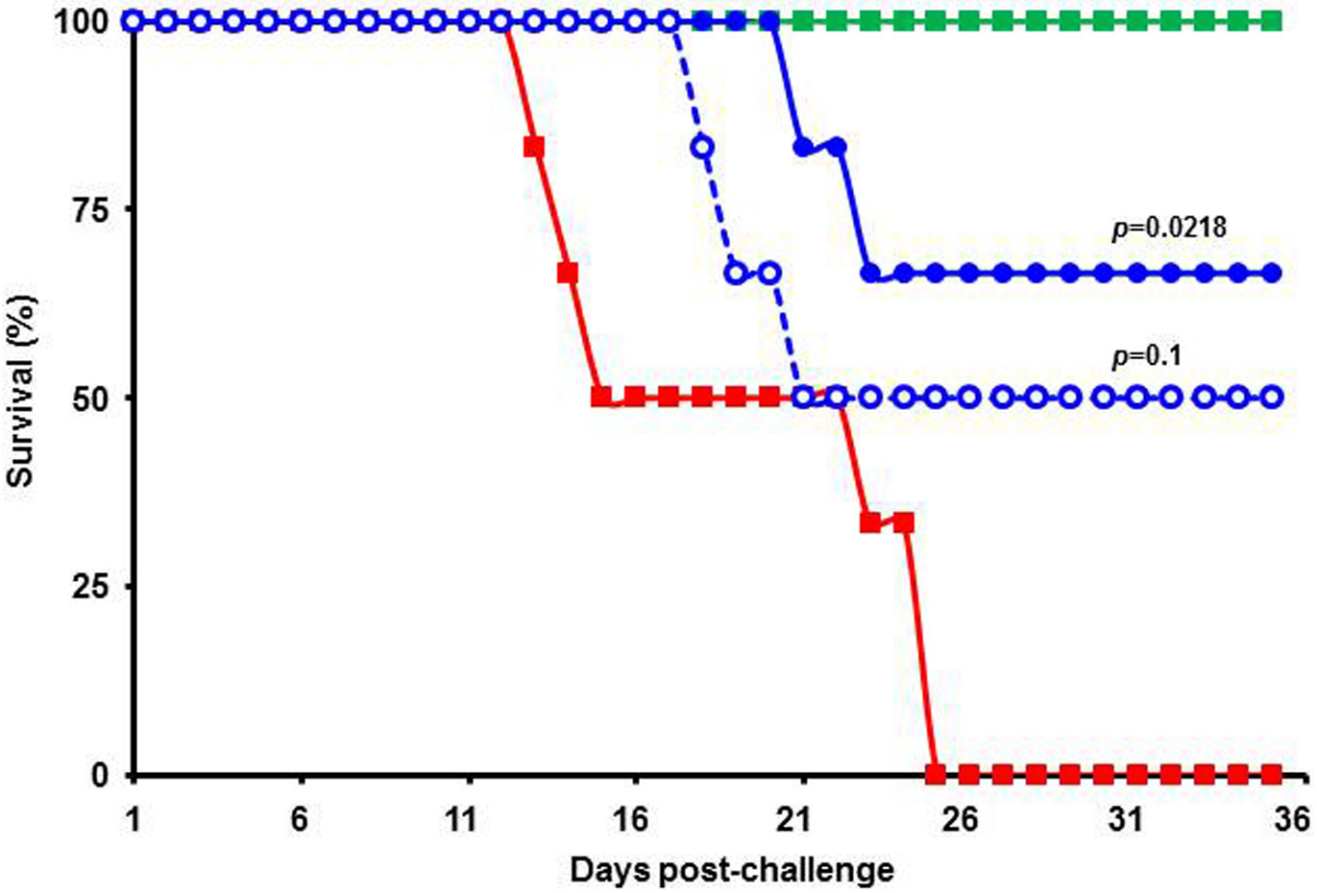
Evaluation of protective efficacy of *Cipa* extract *in vivo*. AG129 mice (9–12 weeks old) were injected i.p. with 10^6^ PFU brain-passaged DENV-2. Infected mice were treated orally with 0.25% methyl cellulose (solid red squares) or *Cipa* extract at 125 mg (empty blue circles) and 250 mg/kg body weight (solid blue circles). Treatment was twice daily for the first 5 days. A sham control group that was not virus-infected, but which received the higher dose of *Cipa* extract orally (solid green squares), was tested in parallel. The mice were monitored daily for mortality and the resultant data plotted as Kaplan-Meier survival curves. The *p* values to assess the statistical significance in the survival rates on day 35 between the *Cipa* extract-treated and placebo-treated (0.25% methyl cellulose) groups were determined using the Log-rank test.

Next, we tested *Cipa* extract for its efficacy *in vivo*. For this, we used the AG129 mouse which has emerged in recent years as a promising dengue model [21, 27] for testing potential DENV inhibitors [28]. This mouse is capable of hosting replication of brain-adapted DENV administered intra-peritoneally (i.p.) and succumbs to it at high challenge doses [27]. We developed a mouse brain-passaged DENV-2 (New Guinea C)-derived challenge strain [22]. At a dose of 10^6^ PFU (given i.p.), it was lethal to AG129 mice, killing them 25 days post-challenge. The illness which preceded death was characterized by ruffled fur, lethargy, hunched back and hind limb paralysis (S2 Fig). We found that the median survival time (MST) of challenged mice treated orally with *Cipa* extract (methanol-free, see ‘Methods‘) twice a day for 5 days post-challenge was increased in a dose-dependent manner. The survival data are present in Fig 4. The MST of challenged mice was 19 days under the experimental conditions. At 125 mg dose, given twice a day for 5 days, survival was 50% and MST was 28 days (*p* = 0.1). This increased to ~67% when the dosage was doubled. Compared to the placebo-treated (0.25% methyl cellulose) group, the level of protection afforded by 250 mg/Kg dose was statistically significant (*p* = 0.021). It is to be ascertained in future if the protective efficacy may be further enhanced by extending the drug treatment regimen beyond 5 days.

### Clinical relevance of *Cipa* extract as a DENV antiviral

**Fig 5:**
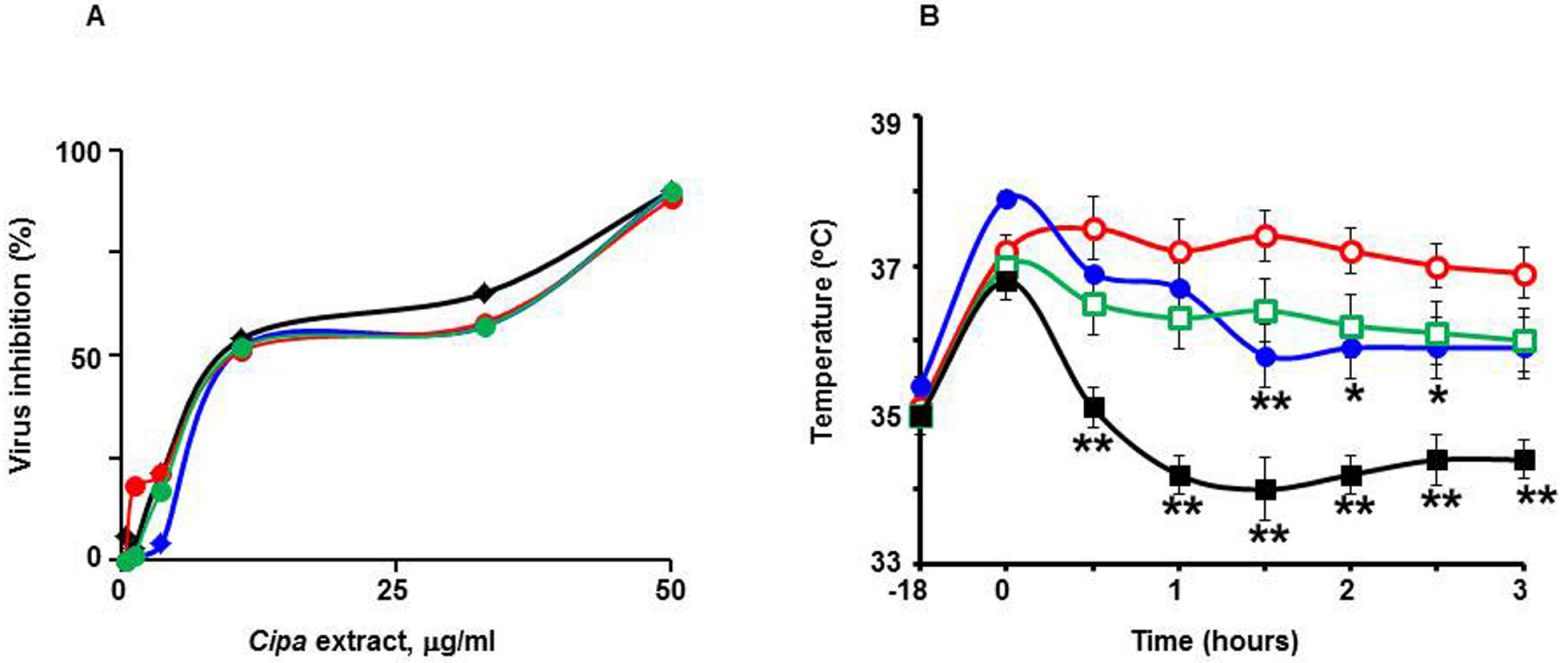
Analysis of interaction between paracetamol and *Cipa* extract. (A) DENV-3 (50 PFU) was pre-incubated with *Cipa* extract in the absence (solid black diamonds) or presence of 1 μg/ml (solid blue diamonds), 10 μg/ml (solid red circles), or 100 μg/ml (solid green circles) paracetamol overnight at 4°C, followed by analysis of viral inhibition in a type-1 assay. (B) Febrile Wistar rats were mock-treated (empty red circles), or treated with paracetamol (solid blue circles), *Cipa* extract (empty green squares) or a combination of both (solid black squares), followed by monitoring of rectal temperature for 3 hours post-treatment at regular intervals. Rectal temperatures between the control (mock-treated) and treatment groups were compared using one-way ANOVA followed by Dunnett’s multiple comparison test (the single and double stars indicate significant differences in the treatment groups with respect to the control group, corresponding to *p* values of ≤0.05 and ≤0.01, respectively).

Since the data so far showed that *Cipa* extracts have potent pan-DENV inhibitory activity, it was considered worthwhile to explore the feasibility of its therapeutic use. As DF is normally treated with paracetamol, it would be important to ascertain the nature of any interaction between *Cipa* and this drug. Dengue disease predisposes some patients to hemorrhagic manifestations and tends to be associated with lowered platelet counts. In this context, it also becomes important to assess if *Cipa* would have any effect on RBCs and platelets. These concerns were addressed in the following experiments. The data from the studies on *Cipa* extract and paracetamol are depicted in Fig 5. A type-1 assay was carried out in which DENV-3 was pre-incubated with serial dilutions of a *Cipa* extract. It was observed that DENV-3 infectivity was inhibited progressively as the *Cipa* extract concentration increased, with an IC_50_ value of 6.1μg/ml. The addition of up to 100μg/ml paracetamol into the DENV-3/*Cipa* extract pre-incubation mix did not significantly affect the inhibitory profile of *Cipa*. The calculated IC_50_ values in the presence of paracetamol at 1, 10 and 100μg/ml were, respectively, 8.4, 7.4 and 8.5μg/ml (Fig 5A). Paracetamol by itself at all concentrations tested did not have any effect on DENV infectivity (plaque counts obtained with DENV-3 alone and DENV-3 plus paracetamol at 100μg/ml were, 43±3 and 45±4, respectively; n = 3). The next experiment examined the effect of *Cipa* extract on the antipyretic activity of paracetamol using the Wistar rat pyrexia model. Interestingly, this experiment revealed that *Cipa* extract possessed intrinsic antipyretic effect (Fig 5B). When rats, in which fever was induced by subcutaneous injection of brewer’s yeast, were treated with *Cipa* extract, the fever was suppressed at an efficiency that was comparable to that of paracetamol. Interestingly, co-administration of *Cipa* extract with paracetamol had a synergistic effect, resulting in a more pronounced decrease in body temperature.

**Fig 6:**
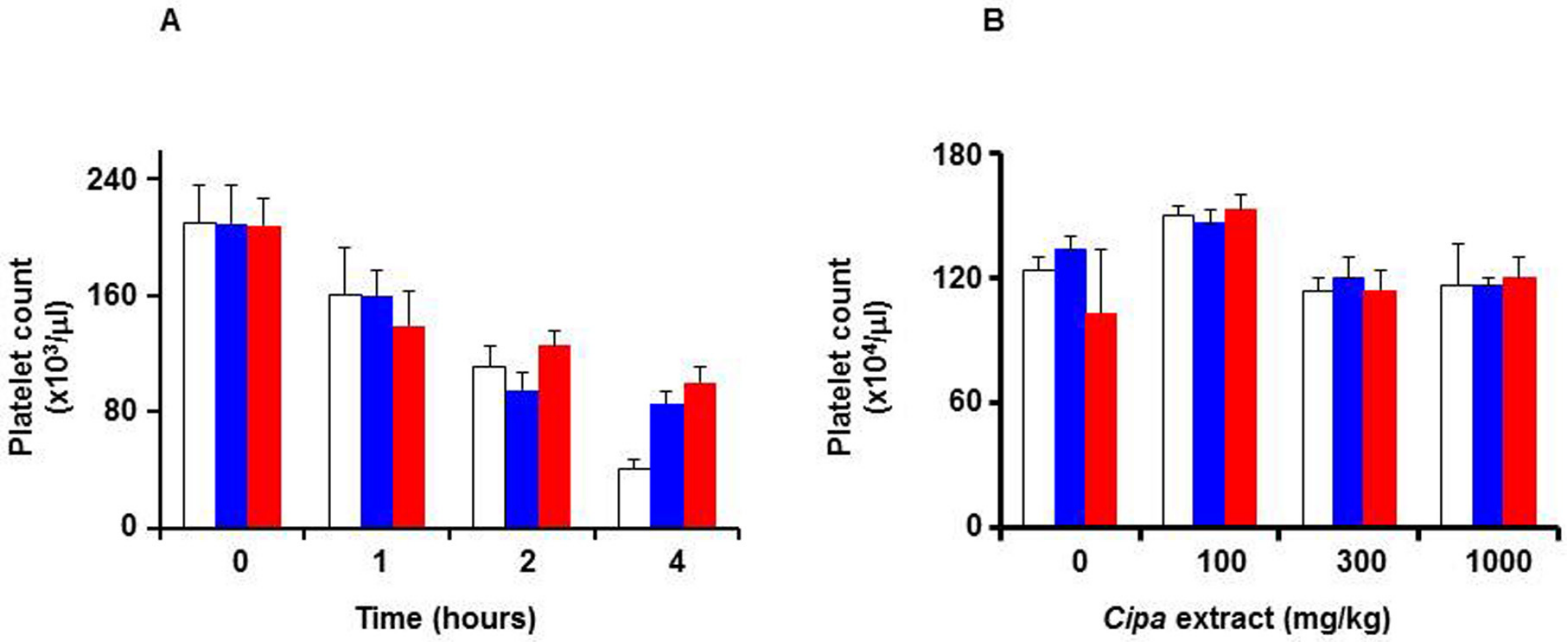
Effect of *Cipa* extract on platelets. (A) Freshly collected human blood was incubated with saline (white bars) or *Cipa* extract (at 2 μg/ml: blue bars; or 10 μg/ml: red bars) for up to 4 hours. Aliquots were drawn at the indicated times for determination of platelet counts. (B) Wistar rats were orally administered 0.25% methyl cellulose containing *Cipa* extract ranging from 0–1000 mg/Kg body weight. Fresh blood collected from these rats at 0 (white bars), 1 (blue bars) and 4 (red bars) hours post-administration, were analysed for platelet counts. For both panels, data shown are mean values (*n* = 5); the vertical bars represent standard deviation, SD.

In the next set of experiments, whole blood from human volunteers was collected and platelets counts obtained before and after 1–4 hours post-mixing with *Cipa* extract. The results are shown in Fig 6A. In the control sample, blood mixed with vehicle (saline), platelet counts declined steadily over time. *Cipa* extract-treated blood samples manifested no statistically significant change in platelet counts with respect to their cognate controls, up to 2 hours (*p*>0.05). At four hours, the *Cipa* extract-treated samples displayed significantly higher (*p*<0.05) platelet counts, compared to corresponding saline-treated control. The apparent platelet-protective effect of *Cipa* extract is intriguing and merits further investigation. Essentially similar results were observed at 2 and 10 μg/ml *Cipa* extract concentrations, indicating that *Cipa* extract did not affect platelets adversely. The effect of *Cipa* extract on platelets was also evaluated in an *in vivo* experiment using Wistar rats. In this experiment, platelet counts were determined in blood drawn from rats which had been given *Cipa* extract orally. The results presented in Fig 6B show that up to 4 hours post-treatment, *Cipa* extract (up to 1000 mg/kg body weight), did not affect platelet counts significantly (*p*>0.05 at the highest dose of *Cipa* extract treatment for 4 hours).

**Fig 7:**
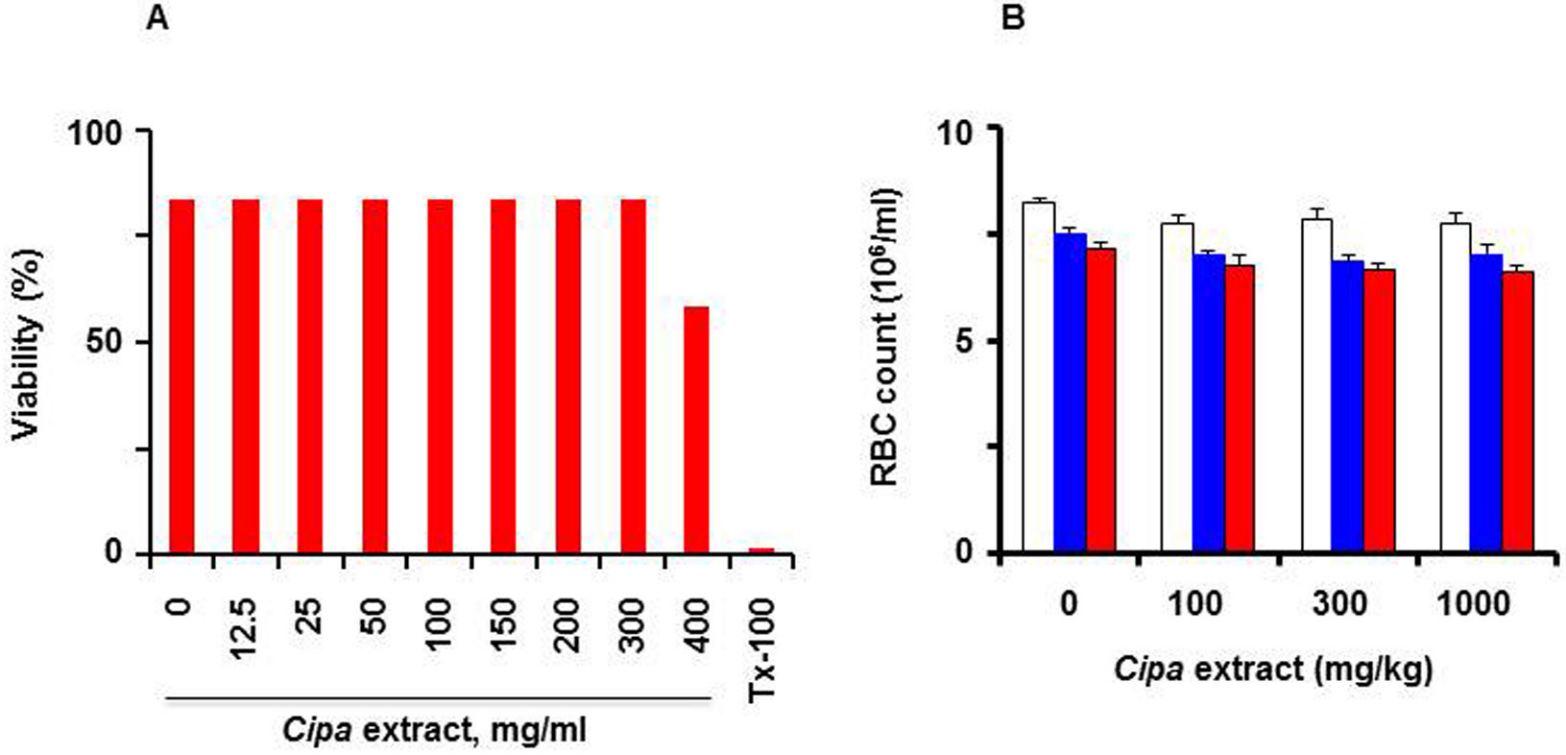
Effect of *Cipa* extract on RBCs. (A) Erythrocytes from freshly collected human blood were incubated for 1 hour at 37°C, with varying concentrations of *Cipa* extract (0–400 μg/ml), followed by measurement of haemolysis at 576 nm. TX-100 represents a control in which an equivalent aliquot of erythrocytes were treated with Triton X-100 to achieve complete lysis. (B) Fresh blood collected from the *Cipa* extract-treated Wistar rats (described in Fig 5B) at 0 (white bars), 1 (blue bars) and 4 (red bars) hours post-administration, were analysed for RBC counts. Data shown are mean values (*n* = 5); the vertical bars represent SD.

The effect of *Cipa* extract on erythrocytes was also assessed, both in *ex vivo* and *in vivo* assays, as done for platelets above. Incubation of freshly collected human erythrocytes with *Cipa* extract at concentrations up to 400 μg/L did not cause discernible haemolysis (Fig 7A). The blood samples, withdrawn from the Wistar rats (given *Cipa* extract, described above), were also analysed for erythrocyte cell counts. This analysis, once again revealed that *Cipa* extract (at concentrations as high as 1000 mg/kg body weight), did not affect erythrocyte counts in the blood of Wistar rats up to 4 hours post-administration (Fig 7B). The difference in erythrocyte counts between the treated and untreated rats was not statistically significant (*p*>0.05). We also analysed total leucocyte and differential counts in the blood of *Cipa*-treated Wistar rats (described in Figs 6B and 7B) and found no significant difference (S3 Fig).

A hallmark of severe dengue disease is the elevation of inflammatory cytokines which are implicated in triggering events that culminate in vascular permeability and haemorrhage [2, 4]. To test if *Cipa* extract had any effect on the secretion of inflammatory cytokines, PBMCs were isolated from blood and incubated with LPS to induce cytokine release. These cells were then treated with *Cipa* extract and the release of pro-inflammatory cytokines monitored using commercial ELISA kits. This experiment showed that the secretion of TNF-α and IL-1β was efficiently suppressed by *Cipa* extract with IC_50_ values of 6.1±1.3 and 5.7±2.7 μg/ml, respectively. An MTT assay showed that at these concentrations, *Cipa* extract has no discernible cytotoxicity in both cell lines tested (CC_50_ = 78.9μg/ml in HepG2; >200μg/ml in LLCMK_2_). These data suggest that *Cipa* extract possesses anti-inflammatory activity in addition to the antipyretic activity documented in the experiment above.

### Preliminary toxicological evaluation

An exploratory toxicological study to assess any adverse effect of repeat dosing over a 1 week span with *Cipa* extract was carried out in Wistar rats. Multiple physiological, haematological, biochemical and clinical parameters were monitored. The results showed that animals treated with up to 2000mg/kg body weight did not manifest any significant changes in any of these parameters compared to vehicle-treated controls (S1–S4 Tables). This essentially corroborates an earlier report that the *Cipa* extract is essentially non-toxic and well tolerated in the animal model [29].

## Discussion

In the absence of a licensed vaccine or antiviral drug, dengue continues to be a significant global public health problem [3, 4]. This is further accentuated in India by its dense human population which represents ~50% of the global population, estimated by WHO to be at risk of dengue. Recently, indigenous diagnostic tests for the early detection of dengue have become available in India [30]. The utility of early detection would be in being able to provide timely antiviral therapy. Further, in the absence of a vaccine, administering antivirals when an outbreak is detected would offer a way of limiting disease spread. While the level of viremia is generally lower in DF, it increases by an order or two in magnitude in DHF/DSS [5, 6]. This observation has led drug developers to hypothesize that lowering viremia by 1–2 logs may be associated with a favourable prognosis [14].

As many of the modern drugs have been derived from natural precursors [17, 18], it is increasingly being regarded that ethnopharmacology and traditional medicines offer an attractive option for identifying starting material for drug discovery initiatives [19, 31]. This provided the basis for the current work which was undertaken to explore the indigenous herbal bio-resource in India to identify plants that may possess anti-DENV inhibitory activity.

Ayurveda represents a distinct discipline in Indian ethnomedicine and has provided a large number of lead molecules for a variety of indications [19]. To provide a rational basis for plant selection, the ayurvedic literature was examined for instances of illnesses with symptoms similar to dengue. Indigenous plants and herbs prescribed for treating these illnesses were chosen for anti-DENV activity screening. In addition plants documented in the literature to possess antiviral activity were also included. As each of the four DENV serotypes can cause severe dengue disease and all of these co-circulate in the hyper-endemic countries, it was considered desirable for a drug to be effective against all the four serotypes. For this purpose a whole cell-based bioassay-guided screening protocol was developed. In principle, an effective antiviral drug could target extracellular virus and block its entry into susceptible cells or act on the virus that has gained entry into cells by targeting one of the post-entry steps in the viral life cycle. Assays were designed to identify herbal extracts targeting both extracellular and intracellular virus. This resulted in the identification of *Cipa* extract and the extract from *Phyllanthus amarus* as possessing pan-DENV inhibitory activity. As the finding of antiviral activity associated with *Cipa* (SI>45) was a novel one, further work focused on it. Its ability to inhibit DENV in a time and dose-dependent manner in the type-1 assay format suggested that the *Cipa* extract possesses a virucidal effect. Further, it was effective as an inhibitor over a ten-fold range of DENV titers suggesting its potential to counteract high viremia. Consistent with this, an analysis of the effect of *Cipa* extract on virus titers demonstrated a >1 log reduction compared to untreated virus controls suggesting its potential utility in altering the course of severe dengue disease to a more favourable outcome. Importantly, *Cipa* extract manifested statistically significant protective efficacy in the AG129 mouse model [21, 27], which has emerged recently as the only promising small animal model for *in vivo* DENV inhibitor testing [28].

At this juncture, two options in terms of future course of development could be envisaged. One, the *Cipa* extract could facilitate the drug discovery process. Two, the *Cipa* extract could be the starting point for developing a carefully standardized herbal formulation for human use. The former option would entail a systematic fractionation to isolate and characterize the active ingredient which may provide small molecule drug lead(s) for optimization and further development. This is both expensive and time-consuming. Additionally, there is the attendant possibility of loss of activity upon fractionation to isolate pure compounds. Considering that *Cipa* is in use for treating human ailments in Ayurvedic medicine in India [32, 33] and in traditional medicine in several countries in South America [34], pursuing the latter option could be a lot simpler and affordable, and of practical utility, particularly in the context of the resource-poor dengue-endemic countries. Preliminary *in vitro* and *in vivo* experiments addressing the clinical relevance of *Cipa* extract support the feasibility of pursuing the second option. For example the drug paracetamol used commonly to treat dengue patients, does not affect the antiviral efficacy of *Cipa* extract. Interestingly, *Cipa* extract appeared to possess an intrinsic antipyretic activity which could synergize with that of paracetamol in the Wistar rat model. It also possessed the ability to down-regulate the secretion of pro-inflammatory cytokines, particularly TNF- α implicated in the pathogenesis of severe disease [2]. Further, *Cipa* extract did not have any discernible effect on platelet counts or on erythrocyte viability. Importantly, the extract was not associated with any adverse toxicology. Finally, another factor in favour of the second option is that, *Cipa* plant is available in several parts of the country and extracts prepared using plants from different geographical locations are fairly similar in terms of their gross overall composition. This suggests that availability is not an issue and extract preparation can be reproducible.

In conclusion, Ayurveda knowledge-based selection of medicinal plants in conjunction with a bioassay-guided screening protocol has resulted in the identification of *Cipa* extract which manifests potent antiviral activity against all four prevalent DENV serotypes. In addition, this extract also manifested dose-dependent protective efficacy in an *in vivo* model, and appeared to be compatible with future clinical use. The outcome of these studies should hopefully pave the way to carry out a systematic development of *Cipa* extract to enable filing of an investigational new drug application with the Drug Controller General of India. Parts of this work have been the subject of patent applications, several of which have been granted [35, 36].

## Supporting Information

S1 Fig Liquid chromatography/mass spectrometry profiles of *Cipa* extracts. Methanolic extracts prepared from *Cipa* plants obtained from Madhya Pradesh (upper panel) and South India (lower panel) were analysed by LC/MS. Each peak is identified by molecular mass (lower number) and retention time (upper number).
(TIF)

S2 Fig Healthy and DENV-2-infected AG129 mice. Panel ‘*a*’ shows a pair of healthy AG129 mice. Panels ‘*b*’ and ‘*c*’ show DENV-2 infected mice manifesting ruffled fur, hunched back and hind limb paralysis.
(TIF)

S3 Fig Total and differential leukocyte counts in *Cipa*-treated Wistar rat blood. (A) Wistar rats were orally administered 0.25% methyl cellulose containing *Cipa* extract ranging from 0–1000 mg/Kg body weight. Fresh blood collected from these rats at 0 (white bars), 1 (blue bars) and 4 (red bars) hours post-administration, were analysed for total leukocyte counts. (B) Blood samples collected from the different groups of Wistar rats described in panel in ‘A’ (vehicle group: white bars; 100 mg *Cipa* group: blue bars; 300 mg *Cipa* group: red bars; 1000mg *Cipa* group: green bars) at the 0 hour time point were analysed for relative proportions of the different leukocytes, presented as percent differential count with respect to the different cell populations (Lym: lymphocytes; Neu: neutrophils; Mon: monocytes; Eos: eosinophils; Bas: basophils; LUC: large unstained cells). (C) Similar data corresponding to those shown in panel B, but generated using the 1 hour blood samples of panel ‘A’. (D) Similar data corresponding to those shown in panel B, but generated using the 4 hour blood samples of panel ‘A’. For all panels, data shown are mean values (*n* = 5); the vertical bars represent SD.
(TIF)

S1 Table Mean body weight and food intake of Wistar rats treated with *Cipa* extract. (DOCX)

S2 Table Hematology parameters in *Cipa* extract-treated Wistar rats. (DOCX)

S3 Table Biochemical parameters in *Cipa* extract-treated Wistar rat sera. (DOCX)

S4 Table Organ weights in *Cipa* extract-treated Wistar rats. (DOCX)
