# Supplementary figures and images for: Cissampelos pareira Linn: Natural Source of Potent Antiviral Activity against All Four Dengue Virus Serotypes
Source: PLoS Negl Trop Dis. 2015 Dec 28;9(12):e0004255. doi: 10.1371/journal.pntd.0004255 (PMC4692392; doi:10.1371/journal.pntd.0004255)

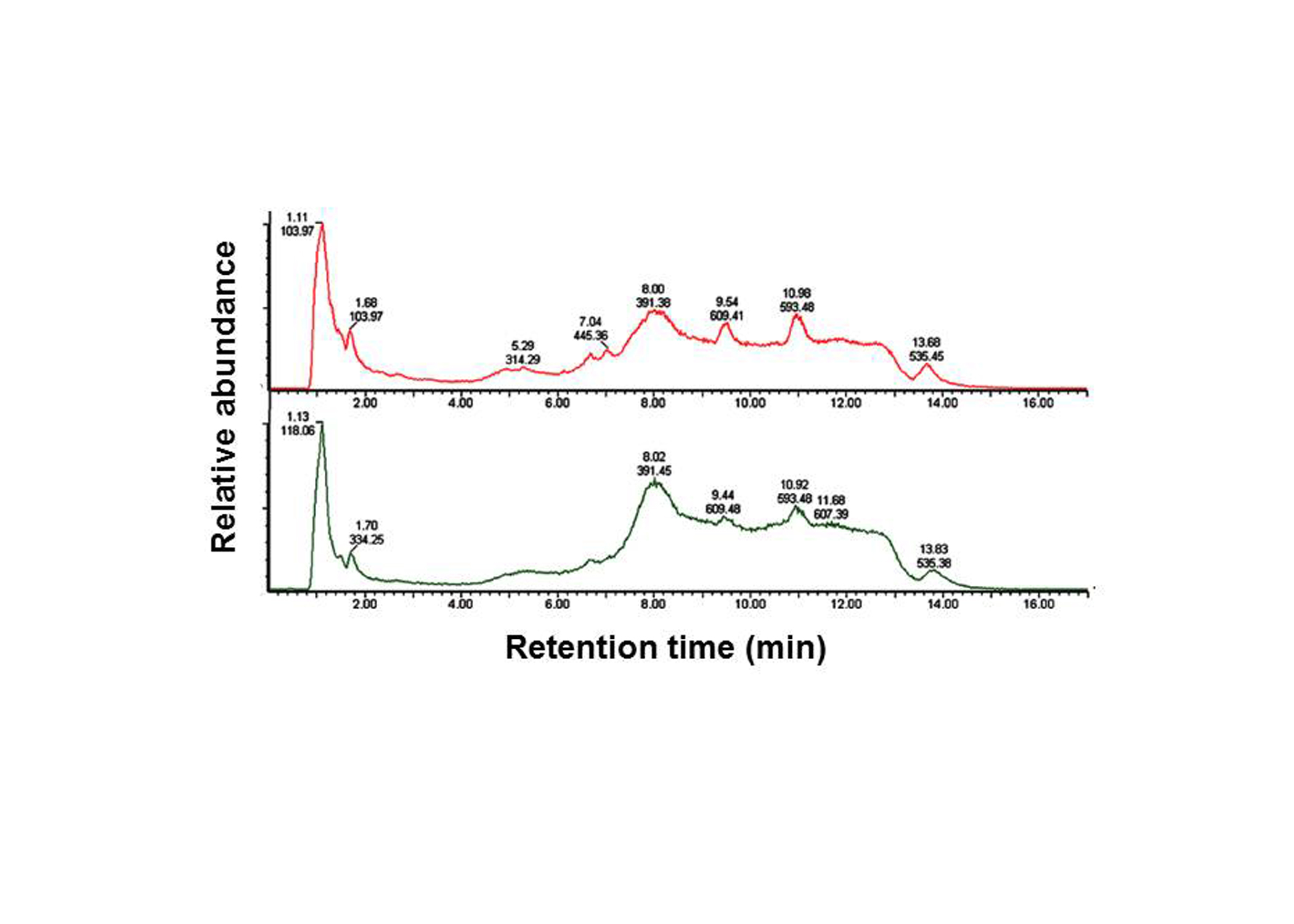

Supplement: S1 Fig — Methanolic extracts prepared from Cipa plants obtained from Madhya Pradesh (upper panel) and South India (lower panel) were analysed by LC/MS. Each peak is identified by molecular mass (lower number) and retention time (upper number). (TIF) [file pntd.0004255.s001.tif]

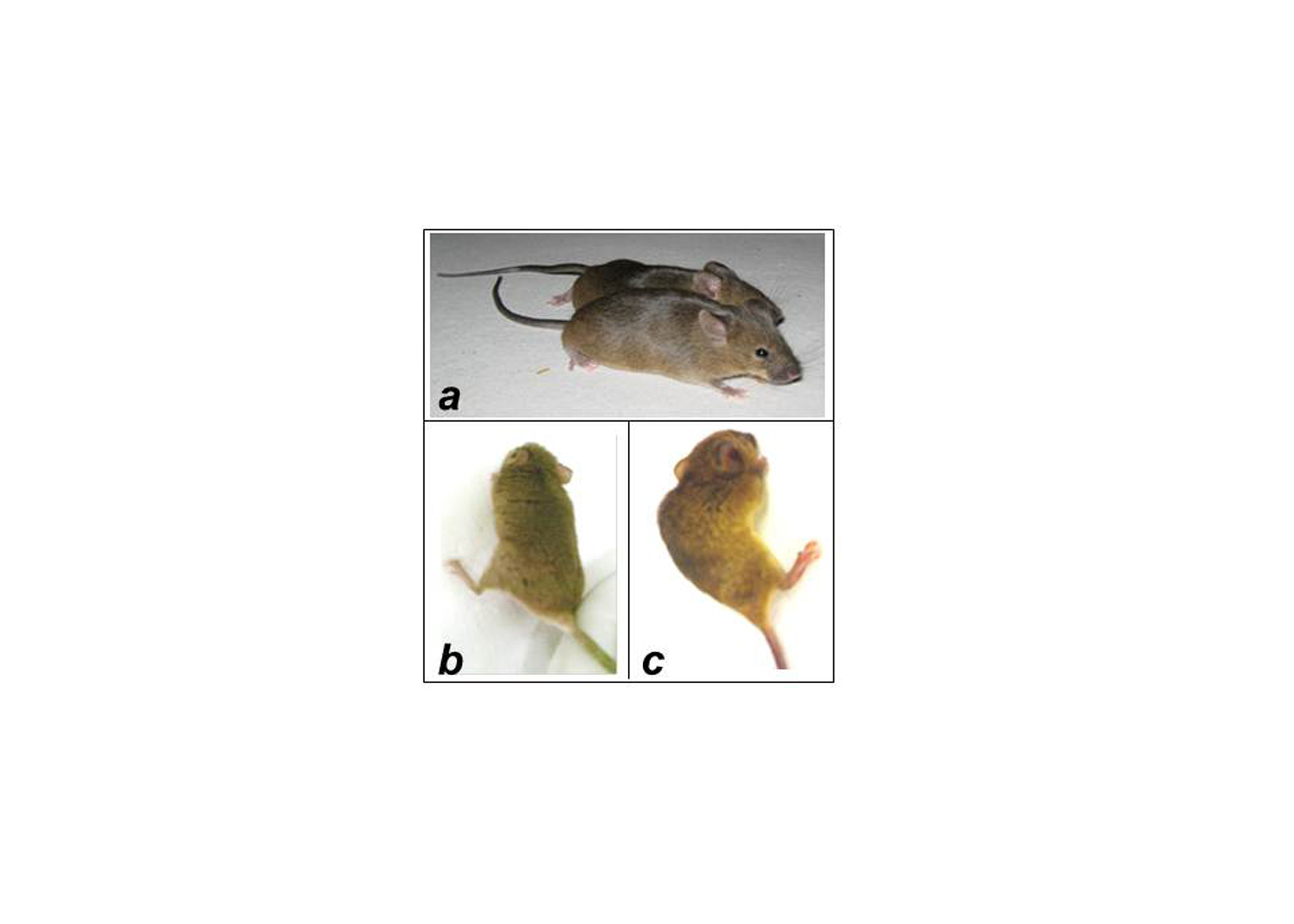

Supplement: S2 Fig — Panel ‘a’ shows a pair of healthy AG129 mice. Panels ‘b’ and ‘c’ show DENV-2 infected mice manifesting ruffled fur, hunched back and hind limb paralysis. (TIF) [file pntd.0004255.s002.tif]

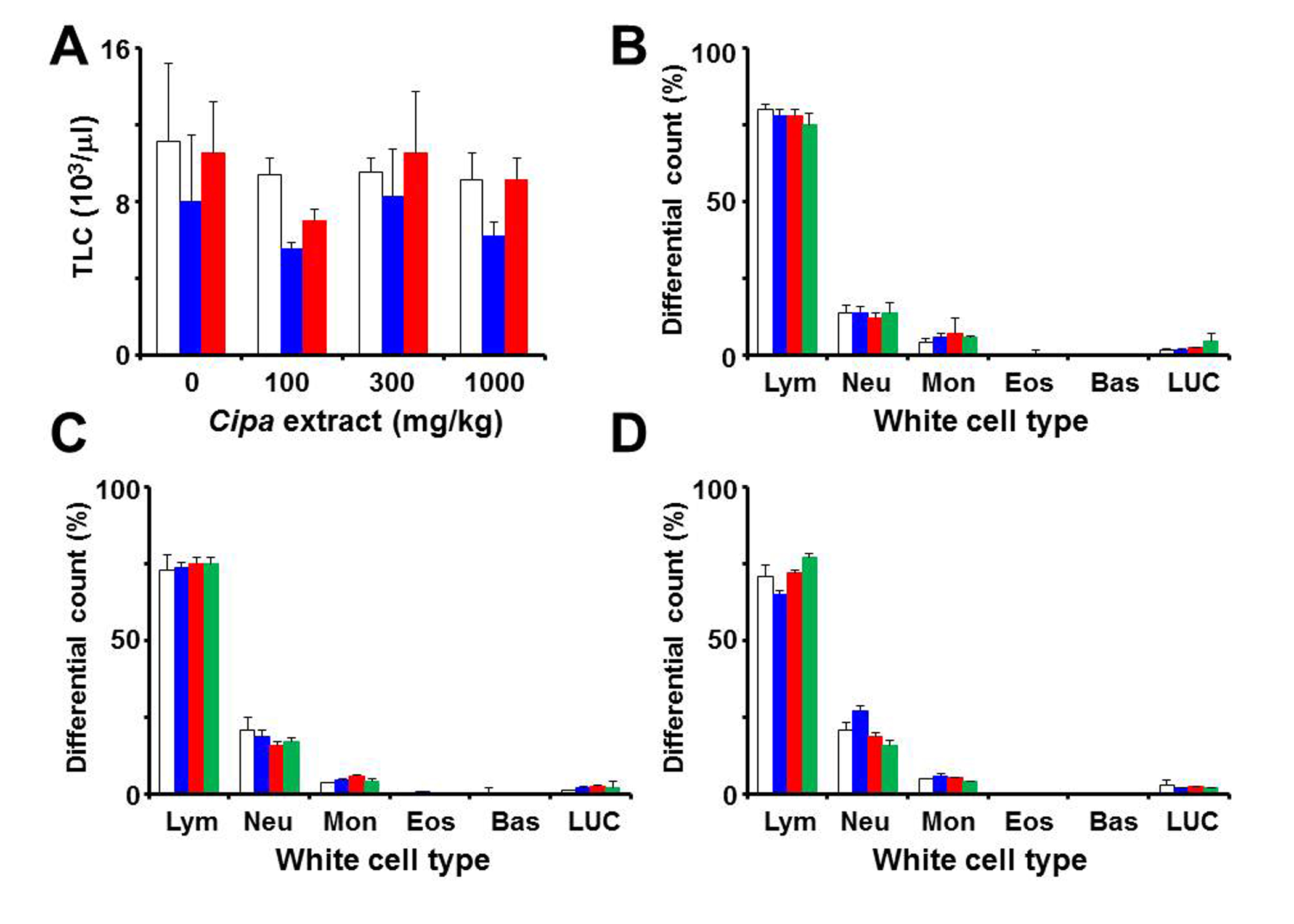

Supplement: S3 Fig — (A) Wistar rats were orally administered 0.25% methyl cellulose containing Cipa extract ranging from 0–1000 mg/Kg body weight. Fresh blood collected from these rats at 0 (white bars), 1 (blue bars) and 4 (red bars) hours post-administration, were analysed for total leukocyte counts. (B) Blood samples collected from the different groups of Wistar rats described in panel in ‘A’ (vehicle group: white bars; 100 mg Cipa group: blue bars; 300 mg Cipa group: red bars; 1000mg Cipa group: green bars) at the 0 hour time point were analysed for relative proportions of the different leukocytes, presented as percent differential count with respect to the different cell populations (Lym: lymphocytes; Neu: neutrophils; Mon: monocytes; Eos: eosinophils; Bas: basophils; LUC: large unstained cells). (C) Similar data corresponding to those shown in panel B, but generated using the 1 hour blood samples of panel ‘A’. (D) Similar data corresponding to those shown in panel B, but generated using the 4 hour blood samples of panel ‘A’. For all panels, data shown are mean values (n = 5); the vertical bars represent SD. (TIF) [file pntd.0004255.s003.tif]
