# Supplementary material for: Cissampelos pareira Linn: Natural Source of Potent Antiviral Activity against All Four Dengue Virus Serotypes
Source: PLoS Negl Trop Dis. 2015 Dec 28;9(12):e0004255. doi: 10.1371/journal.pntd.0004255 (PMC4692392; doi:10.1371/journal.pntd.0004255)
Supplement: S1 Table — (DOCX) [file pntd.0004255.s004.docx]

**S1 Table: Mean body weight***^a^* **and food intake***^b^* **of Wistar rats treated with *Cipa* extract***^c^*

| **Group*^d^*** | **Day 0** | **Day 1** | **Day 2** | **Day 3** | **Day 4** | **Day 5** | **Day 6** | **Day 7** |
| --- | --- | --- | --- | --- | --- | --- | --- | --- |
| Vehicle*^e^* | 210.6 | 213.4 (73.5) | 215.4 (100.2) | 217.2 (92.0) | 220 (99.2) | 222 (93.0) | 224 (103.4) | 226 (101.1) |
| *Cipa*-400 | 213.8 | 215.8 (117.9) | 218.8 (83.2) | 219 (91.0) | 224 (106.4) | 228 (114.0) | 231 (119.2) | 230 (118.2) |
| *Cipa*-2000 | 213 | 216.6 (96.3) | 216.4 (80.6) | 217.4 (83.6) | 220 (93.7) | 225.6 (99.0) | 225.8 (97.0) | 226 (92.4) |

*^a^*Body weight in g

*^b^*Food intake in mg (starting from day 1), shown in parentheses

*^c^*Administered orally (as gavage, using oral feeding needle), once daily, for 7 days

*^d^*5 animals per treatment group (Vehicle:4 ml 0.25% methyl cellulose/kg body weight; *Cipa*-400 and *Cipa*-2000: 4 ml vehicle containing 400mg and 2000mg *Cipa* extract/kg body weight, respectively)

*^e^*0.25% methyl cellulose, 8 ml/kg body weight
