# Supplementary material for: Cissampelos pareira Linn: Natural Source of Potent Antiviral Activity against All Four Dengue Virus Serotypes
Source: PLoS Negl Trop Dis. 2015 Dec 28;9(12):e0004255. doi: 10.1371/journal.pntd.0004255 (PMC4692392; doi:10.1371/journal.pntd.0004255)
Supplement: S2 Table — (DOCX) [file pntd.0004255.s005.docx]

**S2 Table: Hemaotology parameters***^a^* **in *Cipa* extract-treated Wistar rats**

| **Group*^b^*** | **Time*^c^*** | **WBC (x10^9^/L)** | **RBC (x10^12^/L)** | **Hemoglobin (g/dl)** | **HCT*^d^* (%)** | **Platelets (x10^9^/L)** |
| --- | --- | --- | --- | --- | --- | --- |
| Vehicle | B | 9.5±0.80 | 7.71±0.48 | 14.6±0.8 | 39.5±2.1 | 514.8±26 |
|  | A | 7.1±1.0 | 6.6±0.27 | 13.2±0.3 | 36.6±1.1 | 644.0±41 |
| *Cipa*-400 | B | 10.7±1.1 | 7.03±0.09 | 13.7±0.3 | 37.4±0.5 | 533.0±41 |
|  | A | 8.7±0.8 | 6.2±0.15 | 12.5±0.4 | 34.5±0.9 | 605.0±37 |
| *Cipa*-2000 | B | 11.9±0.9 | 7.74±0.28 | 14.8±0.4 | 40.1±1.0 | 532.0±15 |
|  | A | 9.1±0.5 | 6.9±0.22 | 14.9±1.0 | 37.4±1.0 | 623.0±18 |

*^a^*Values shown are mean±SE

*^b^*Groups are as in S1 Table

*^c^*Time of sample collection either before initiating (B) or after (A) 7 days of *Cipa* extract treatment

*^d^*Hematocrit
