# Supplementary material for: Cissampelos pareira Linn: Natural Source of Potent Antiviral Activity against All Four Dengue Virus Serotypes
Source: PLoS Negl Trop Dis. 2015 Dec 28;9(12):e0004255. doi: 10.1371/journal.pntd.0004255 (PMC4692392; doi:10.1371/journal.pntd.0004255)
Supplement: S3 Table — (DOCX) [file pntd.0004255.s006.docx]

**S3 Table: Biochemical parameters***^a^* **in *Cipa* extract-treated Wistar rat sera**

| **Group*^b^*** | **Time*^c^*** | **SGOT**  **(IU/l)** | **SGPT**  **(IU/l)** | **Creatinine**  **(mg/dl)** | **Glucose**  **(mg/dl)** | **Cholesterol**  **(mg/dl)** | **Total protein**  **(g/l)** | **Albumin**  **(g/dl)** | **Urea**  **(mg/dl)** |
| --- | --- | --- | --- | --- | --- | --- | --- | --- | --- |
| Vehicle | B | 166.8 | 56.56 | 0.69 | 99.3 | 248.3 | 10.5 | 6.3 | 44.4 |
|  | A | 166.3 | 53.2 | *nd* | 117.8 | 249.6 | 9.6 | 3.3 | *nd* |
| *Cipa*-400 | B | 164.7 | 50.2 | 0.57 | 105.5 | 243.8 | 11.3 | 5.8 | 34.4 |
|  | A | 162.7 | 58 | *nd* | 107 | 252 | 9.3 | 3.2 | *nd* |
| *Cipa*-2000 | B | 152.7 | 49.7 | 0.54 | 126.5 | 250.9 | 12 | 5.5 | 30 |
|  | A | 152.5 | 44.2 | *nd* | 95.4 | 254.1 | 9.2 | 3.4 | *nd* |

*^a^*Values shown are mean

*^b^*Groups are as in S1 Table

*^c^*Time of sample collection either before initiating (B) or after (A) 7 days of *Cipa* extract treatment

*nd*: not done
