# Supplementary material for: Cissampelos pareira Linn: Natural Source of Potent Antiviral Activity against All Four Dengue Virus Serotypes
Source: PLoS Negl Trop Dis. 2015 Dec 28;9(12):e0004255. doi: 10.1371/journal.pntd.0004255 (PMC4692392; doi:10.1371/journal.pntd.0004255)
Supplement: S4 Table — (DOCX) [file pntd.0004255.s007.docx]

**S4 Table: Organ weights***^a^* **in *Cipa* extract-treated Wistar rats**

| **Group*^b^*** | **Sex** | **Body weight** | **Brain** | **Thymus** | **Heart** | **Liver** | **Spleen** | **Kidney** | **Adrenal** | **Gonad** |
| --- | --- | --- | --- | --- | --- | --- | --- | --- | --- | --- |
| Vehicle | M | 212.5 | 1.698 | 0.276 | 0.785 | 8.751 | 0.895 | 0.717 | 0.019 | 1.368 |
|  | F | 198 | 1.560 | 0.215 | 0.715 | 7.030 | 1.041 | 0.565 | 0.030 | 0.077 |
| *Cipa*-400 | M | 211.3 | 1.619 | 0.261 | 0.734 | 8.354 | 0.735 | 0.715 | 0.020 | 1.326 |
|  | F | 186.5 | 1.639 | 0.181 | 0.718 | 6.903 | 0.652 | 0.595 | 0.025 | 0.050 |
| *Cipa*-2000 | M | 198 | 1.737 | 0.267 | 0.751 | 9.136 | 0.765 | 0.635 | 0.020 | 1.202 |
|  | F | 195 | 1.775 | 0.199 | 0.771 | 7.427 | 0.792 | 0.674 | 0.025 | 0.055 |

*^a^*Values shown are in g (wet weight)

*^b^*Groups are as in S1 Table
